# Type, Content, and Source of Social Support Perceived by Women during Pregnancy: Evidence from Matlab, Bangladesh

**DOI:** 10.3329/jhpn.v29i2.7859

**Published:** 2011-04

**Authors:** Joyce K. Edmonds, Moni Paul, Lynn M. Sibley

**Affiliations:** ^1^Center for Research on Maternal and Newborn Survival, Emory University, Atlanta, GA, USA; ^2^Reproductive Health Unit, Public Health Sciences Division, ICDDR, B, GPO Box 128, Dhaka 1000, Bangladesh; ^3^Center for Research on Maternal and Newborn Survival, Emory University, Atlanta, GA, USA

**Keywords:** Cross-sectional studies, Pregnancy, Qualitative studies, Reproductive health, Retrospective studies, Social support, Stress, Bangladesh

## Abstract

Specific and contextualized data on social support during distinct health events are needed to improve social support interventions. This study identified the type, content, and source of social support perceived by women during pregnancy. In-depth interviews with 25 women, aged 18-49 years, living in Matlab, Bangladesh, were conducted. The findings demonstrated that women perceived, the receipt of eight distinct types of support. The four most frequently-mentioned types included: practical help with routine activities, information/advice, emotional support and assurance, as well as the provision of resources and material goods. Sources varied by type of support and most frequently included-—mothers, mothers-in-law, sisters-in-law, and husbands. Examples depicting the content of each type of support revealed culturally-specific issues that can inform community-based social support interventions.

## INTRODUCTION

Social support is broadly defined as the resources provided by other persons (1) and can be conceptualized as the function of one's network (2, 3). Social support is considered one of the mechanisms through which social networks are thought to affect health (4). Yet, social support is not a uni-dimensional, fixed set of resources, rather it is situation-, issue and context-specific (5, 6). There is a need to operationally define the concept of social support in a way that adheres to meanings prescribed by people with direct experience of the phenomena under study. Thus, qualitative work that explores social support in distinct cultural settings during specific life-events is an important first step in elucidating the influence of social support networks on health behaviours and subsequent outcomes (7). However, research on social support has overwhelmingly focused on developing countries in a Western context employing generic measures that, despite their psychometric properties, lack relevance to a particular population (3). Little is known about the precise nature of social support in South-East Asian contexts during pregnancy. This paper presents findings of a qualitative study designed to explore the specific dyadic characteristics (type, content, and source) of women's perceived social support during pregnancy in Matlab, Bangladesh.

Pregnancy is typically considered a vulnerable period for women. One important risk factor affecting maternal well-being is lack of social support (8). Low social support is associated with low birthweight (9, 10), poor labour progress (11), preterm labour (8, 12), neural tube defects (13), depression and anxiety (11, 14). In addition, social support networks affect pregnancy-related health behaviours and lifestyle habits, including use of prenatal and delivery services and dietary habits (15, 16). The association between social support and pregnancy outcomes is complex, involving the psychological and biological (i.e. hormones and immune mediators) response to life-events and stress.

Overall, the evidence suggests that social support is protective during pregnancy. Pregnant women need the support of family members, friends, and health professionals. However, two systematic Cochrane reviews reported no benefit of interventions providing ‘additional’ support during pregnancy either for reducing premature birth (17) or for preventing postpartum depression (18). One explanation for these negative findings is that the intervention trials did not account for the functional, qualitative aspects of social support that are grounded in experience or a specific context (8). The interventions may not have provided the needed type of support by available, accessible and trustworthy individuals who share common experiences. Wellman suggests that scholars interested in the implications of social support on health “must take into account the varied nature of social support and of those who provide it” (19). He contends that investigators should consider the aetiology of support and seek a more fine-grained understanding of the type of relations that produce particular kinds of support. The development of culturally-tailored interventions depends on the specificity of such data. For example, Norbeck *et al.* based their effective social support intervention to prevent low birthweight among African American pregnant women on qualitative work that identified a woman's mother and male partner as critical sources of emotional support (20). As a first step in enhancing the relevance and validity of social support interventions, this study sought to identify and characterize the functions of social support during pregnancy as perceived by women in a developing-country setting.

## MATERIALS AND METHODS

### Study site

The study was conducted in Matlab, Bangladesh, a rural subdistrict located 55 km southeast of the capital city Dhaka. The vast majority of the population is Muslim, and the remainder is Hindu, Buddhist or Christian. Islam is the main religion, and almost all residents speak in Bangla, the official national language. The principal economic activities are agriculture and fishing. Remittances from men who migrate to the city or abroad for work opportunities are another source of income for households in the area. The primary modes of transportation within Matlab are by foot, rickshaw, country boat, or three-wheeled taxi. Patrilineal and virilocal family is the traditional type of family-unit found in rural Bangladesh, although changes to kinship organization are occurring due to the rapid socioeconomic transitions, including employment opportunities in cities and abroad. Kinship networks, however, remain a universal aspect of the social structure, and division of labour is predominantly gender-based in rural villages with exceptional patterns among the poorest and most educated women.

Matlab has a unique maternal healthcare infrastructure due to the renowned efforts of the Maternal and Child Health and Family Planning Programme (MCH-FP) of ICDDR, B serving one half of the Demographic Surveillance System (DSS) area established in the early 1960s—now called Health and Demographic Surveillance System (HDSS). The Matlab health service area (MHSA) currently provides healthcare coverage to approximately 113,660 people, of whom 31,527 are women aged 15-49 years through the ICDDR, B's four health subcentres and town hospital (21). The service area, where the study was conducted, consists of four blocks and 67 villages.

### Methods

A retrospective, cross-sectional research design was used. In-depth, semi-structured interviews using a prepared interview guide were conducted with a non-random, sample of 25 community-dwelling women from the population of interest during August 2008. The population of interest was Bangladeshi women aged 18-49 years residing in the MHSA, who had an uncomplicated pregnancy and delivery, resulting in a livebirth between 26 May 2008 and 10 August 2008. The total sample (n=25) was purposively divided between women who had delivered in the home (n=12) and in a facili-ty (n=13). The sample was selected to be as representative as possible of the population of interest. Collection of data concluded after 25 interviews because the respondents were converging on consensus, and the central limit theorem states that normal approximation is typically good with 25 observations or more. Data collection occurred no more than three months after the index birth. The sample was drawn from the maternal and child programme database and recruited using an active household strategy. This is a process in which members from the eligible population are located and invited to participate in the study by research staff. The staff travel within a community household-by-household assisted by sample rosters stratified at the village level.

### Collection of data

The primary data-collection technique was individual in-depth interviews guided by Spradley's ethnographic interviewing techniques supplemented by participant-observation (22). A bilingual Bangladeshi research officer, who had accrued five years of experience conducting in-depth interviews and focus groups among villages in the study area, directly asked each participant a series of closed and open-ended questions. Close-ended questions asked about sociodemographic and reproductive health characteristics. Open-ended questions asked about the people (relatives) who provided respondents with support during pregnancy. Specific culturally-relevant probes generated responses about the types of support provided by each relative mentioned. Initially, the probes were derived a priori from a widely-cited functional definition of social support by House who defined it as an interpersonal transaction involving one or more of the following: emotional concern, instrumental aid, information, and appraisal (23). The probes evolved as specific examples of support emerged. In the later stages of interviewing, questions were based on the analysis of early information to find contradictory, confirmative and alternative examples. Responses were recorded using a matrix of the most common kin and non-kin relations and their local Muslim and Hindu terms as described in Aziz's seminal work on kinship in Bangladesh (24). A list of relations categorized by type of support was generated after each interview.

One to two interviews were conducted per day. All interviews were conducted in the home. The average duration of the interviews was 70 [standard deviation (SD) 9.9] minutes. The research officer and the Principal Investigator debriefed after each day of interview for approximately 45 minutes per interview. All interviews were audio-taped using an Olympus Digital DS-2300 recorder, and the field notes served as a back-up method of retrieving data. Each interview was transcribed verbatim and translated, assisted by a transcription software. Two research officers, bilingual in Bangla and English conducted the transcription and translation. The research officer who conducted the interviews transcribed these in Bangla; the other one translated the Bangla transcription into English. Finally, the research officer who conducted the interviews verified the completed English transcripts together with the Principal Investigator.

### Analysis of data

Sociodemographic and reproductive health characteristics were analyzed in the SPSS software (version 16.0) using simple frequencies (25). The transcripts were read repeatedly together with the field-note summaries for each individual respondent to extract an initial understanding of the data. Line-by-line content analysis was conducted searching for examples of support functions, which were highlighted and later coded manually. Individual data units (words, phrases, and sentences) representative of support functions were considered from the emic perspective (using the respondent's own words and descriptors to explain the phenome-non) and used for generating codes for categories of support. The data were compared across cases to find confirmations, contrasts, or contradictions. The support categories found were systematically expanded, developed, and summarized with a focus on examples representing the content of each category. The sources of support for each respondent were organized by support category using data from the kinship matrix. Frequency ranking determined the most salient categories of support provided to respondents during their pregnancy. Sources of support for the four most frequently-mentioned support categories were tabulated across interviews according to what a respondent mentioned. Results of the analysis were reviewed and corroborated with the research officer who conducted the interviews.

### Ethical approval

The Institutional Review Board of the Emory University and the Ethical Review Committee of ICDDR, B both approved the study. Verbal informed consent of all women who volunteered to participate was obtained following standard disclosure procedures.

## RESULTS

The characteristics of the sample are presented in Table 1. The mean age of the female participants was 25.7 (SD 6.6) years. All the respondents were married. Ninety-two percent were Muslims, with a Hindu minority (8%). The average educational attainment was 6 (SD 4) grades, indicating completion of primary schooling. The average number of livebirths, including the index birth, was 2.4 (SD 1.3, range 1-6). In response to a question about how well the respondent could read or write, 12 (48%) did so with ease, six (24%) with difficulty, and seven (28%) not at all. Over half (52%) of the women reported not having regular exposure to the media (e.g. watch television or listen to radio at least once a week). Asset scores, a proxy for socioeconomic status, ranged from 1 to 5, with 1 being the lowest wealth quintile and 5 being the highest. The average asset score was 3.3 (SD 1.4). The mean household-size was 7.8 (SD 2.5). The large majori-ty (72%) of the women in the sample lived with the husband and husband's kins, characteristic of patrilocal kinship systems.

**Table 1.** Descriptive characteristics of the study sample (n=25)

Sample characteristics	Total sample	
	No.	%	
Age (years)		
18-21	8	32
22-25	5	20
26-29	4	16
≥30	7	28
Parity		
1	8	32
2	5	28
3	3	20
≥4	8	8
Education attainment		
Never attended	5	20
1-5 grades (primary)	5	20
6-10 grades (secondary)	14	56
11-12 grades	1	4
Literacy		
Easily	12	48
With difficulty	6	24
Not at all	7	28
Regular exposure to media		
Yes	12	48
No	13	52
Religion		
Muslim	23	92
Hindu	2	8
Asset score		
1 (lowest)	3	12
2	5	20
3	5	20
4	7	28
5 (highest)	5	20
Type of household		
Marital	18	72
Nuclear	5	20
Natal	2	8

### Type and content of support

Women perceived eight types of support during their pregnancy period. These included practical help with routine activities, information and advice, emotional support and assurance, resources and material goods, logistic communication, prayer and spiritual rituals, nutritional support, and accompaniment outside the homestead. Specific examples depicting the content of each type of support are presented in Table 2. The four most frequently-mentioned support categories in rank order were: (a) practical help with routine activities, (b) information and advice, (c) emotional support and assurance, and (d) resources and material goods.

**Table 2.** Description of types of support with examples of content

Type of support	Content examples
Practical help with routine activities	Milked cows, prepared fishes
	Cleaned and strained rice
	“Brought water from tubewell for cooking in big pots”
	Fed other children
	Washed clothes
	Went to market when husband not in the home
	Helped with heavy work during harvest season, husking paddy
	Made fire for cooking
Information and advice	“Do not go to roof to hang clothes”
	“When ill, take rest”
	“Give prayer five times a day”
	“Take food properly”
	“Do not work with cold water”
	“Do not stay in field at night because of evil spirits”
	“Keep a match with you when you go out at night”
	“Call husband to do heavy work”
	“Abide by father-in-law and mother-in-law, words they say”
Emotional support and assurance	“Do not worry” *“Chinta koro na”*
	Inquired about how she is feeling
	“Do not be tense”
	Gossiped
	“If you have a problem, I will go with you to facility”
	Talked with her when she feels unsure
	Massaged body with mustard oil
	Tell me if you have any physical problems, inform of discomfort or changes
Monetary and material goods	Paid for urine test to confirm pregnancy
	Bought and delivered medications
	Paid rickshaw fare to clinic
	Paid costs for antenatal care
	Paid *kobiraj* for amulets and rituals
	Saved money in case of complication
Logistic communication	Went out to locate *dai*
	Informed others in household of pregnancy and labour pains
	Phoned mother to come to marital home when labour started
	Went to find rickshaw-puller or country boatman
Prayer and spiritual rituals	Gave holy blow and gave blessed water
	Brought amulets from *kobiraj*
	Brought blessed molasses to increase labour pains
Nutritional support	Gave milk and tea during labour pains
	Brought special foods, such as grapes and guavas, from bazzar upon request
	Made foods she likes when sick
Accompaniment	Accompanied and assisted with bathing in pond
	Accompanied at night to latrine
	Accompanied to facility or met at facility
	Stayed at facility during labour

### Sources of support

In total, 19 unique relations were perceived as sources of support. The average number of supportive relations mentioned was 9.96 (SD 1.9, range 6-15). All the relations were described using descriptive kin terminology, with two exceptions—community health research workers (CHRWs) and neighbours. CHRWs are employed by ICDDR, B and visit households once every two months to collect demographic data and provide health-promotion and preventive services. Neighbours are often extended family living in adjacent *baris* (homesteads). The most frequently-mentioned supportive female kinship relations were: mother, sister, mother-in-law, husband's sister, husband's brothers or cousins and their wives, and wives of husband's uncles. In the Bangladeshi context, wives of husband's brothers or cousins are referred to as *jals*, a kinship term that translates into sister-in-law. The most frequently-mentioned supportive male kinship relations were husband, father, and father-in-law. CHRWs and neighbours were the two most frequently-mentioned supportive non-kin relations.

### Type of support by source

**Fig. 1.**
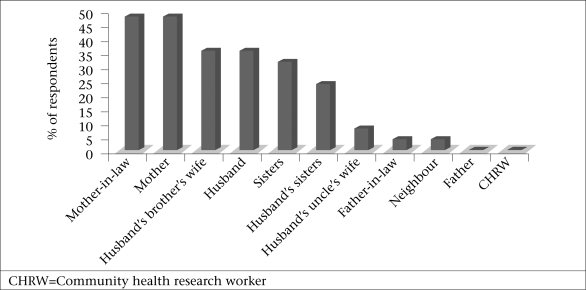
Sources of practical/routine help

**Fig. 2.**
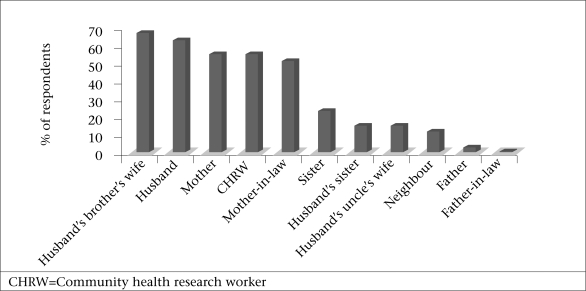
Sour ces of information and advice

**Fig. 3.**
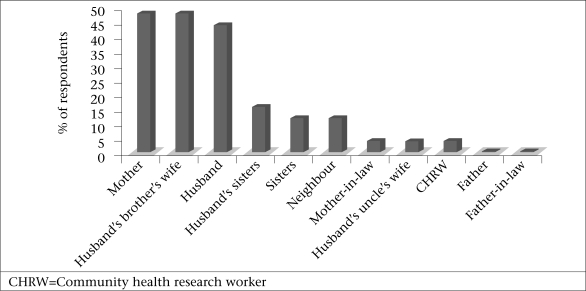
Sour ces of emotional support

**Fig. 4.**
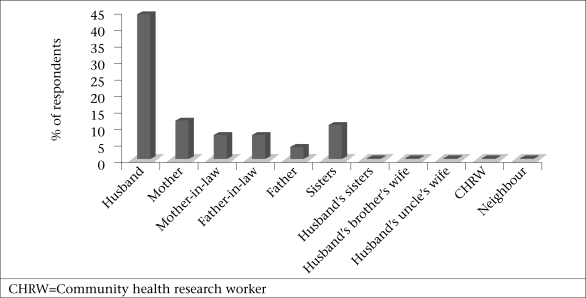
Sources of resources and material goods

The hierarchy of relations varied by type of support provided. The percentages of the respondents reporting the four top ranked types of support by source are shown in Figure 1-4. Responses indicated that mothers, mothers-in-law, and husbands were the most multiplex of relations, providing all four categories of support. Multiplex is a social network term meaning multiple types of support among the same set of people.

During the interviews, support categories were discussed in relation to the type of activities they compromised. De-identified quotations from the transcripts were used for illustrating the content of the top four ranked categories of support and key themes. In the quotes, the words of the respondents are presented in format text and those of the interviewer in bold.

#### Practical help with routine activities

The vast majority of the examples involved practical help with routine activities or the provision of skill, labour and time resources in relation to regularly-performed household duties. The examples were consistent with descriptions of women's production in which rural Bangladeshi women work within or near the household in food processing, food preparation, household maintenance, horticulture and animal husbandry, and childcare (26). They work for long hours, and the work is physically demanding (i.e. carrying water from the well and husking the paddy). Mothers-in-law, mothers, and sisters-in-law were the top three sources of practical help.

She (sister-in-law) did not let me separate cooked rice from the liquid—*vater mar gala*—it is considered a heavy work since the hot rice-pot needs to be lifted and held for sometime. She filled the pitchers—she did everything when I had abdomen pain. (R16)

My husband used to help me with things I could not do. For example, carrying water for cows. (R23)

*Shashuri* (mother-in-law) cooked, filled up pitchers, and she helped. (R23)

**M: “You have two jals (sisters-in-law). How did they help you?”** R: “She helped, helped me in cooking. When she saw me cooking for a long time, she told me to ‘get up ….’ (R01)

**M: “Who used to help you in your daily work?”** R: “There are my mother and *bhabi* (wife of my brother) living in my house. *Bhabi* used to do household works. When I went for a bath, my mother looked after me so that I did not fall down.” (R09)

The impression gleaned from the interviews was that women's level of daily work was reduced when residing in or visiting their natal homes. Reduction of workload was described as a clear incentive for natal home-visits during pregnancy, not just for delivery as traditionally dictated by the sociology of first births in Bangladesh. Pregnant women likely benefit from the close social and physical support that comes with spending time in their natal home, a point that is illustrated in the following quotes:

In my father's house, they did not let me do any work. I ate and slept. They did not even let me hang my mosquito net. My mother or sister did this for me. My sisters also washed clothes for me. (R21)

I did not work in my father's house. My younger sister and mother did all the work. (R22)

When I was in my mother's house, my sisters helped me. They brought for me whatever food I could eat. I used to vomit instantaneously after eating. I could not keep the food inside. They used to prepare what I wanted to eat. (R03)

It was more work during the harvest time (in marital home). I went to my father's house (natal home). (R01)

For one woman, the anticipated level of pregnancy and postpartum support in her natal home influenced the final decision about the place of delivery.

My husband preferred to take me (to deliver) at the (natal) home in village because more care could be provided there. (R01)

#### Information and advice

Information and advice refer to feedback about the women's condition or situation. Sisters-in-law (68%), husbands (64%), mothers (56%), and CHRWs (56%) were the most frequently-mentioned sources of information and advice. Place of delivery was a common topic as evidenced from the following quotes.

She (CHRW) told that home-delivery would be bad. She told me to go to Matlab on the expected date. She told me that it would be good to go to Matlab. If your baby is born in Matlab, it will get good treatment. You and your baby both will be safe and sound. (R19)

She (CHRW) told me, ‘do not do heavy work’. She told me not to go to father's house (natal home) and to stay here. ICDDR, B's hospital is nearby and told me to stay here. (R10)

**M: “You talked with your niece about your comfort and discomfort. What did she say?”** R: She told me to go to ICDDR, B. Here in our area everyone goes to ICDDR, B. Home-delivery does not take place usually. (R11)

Other kinds of advice were more culture-specific and reflect the persistence of traditional practices surrounding mobility during pregnancy. Pregnant women are advised to not go out during the afternoon or evening for fear that they might become possessed by free-ranging spirits thought to cause poor pregnancy outcomes. These evil spirits are believed to catch a woman when she travels alone outside her residence, especially during early morning, noon, and at dusk (27).

She (sister-in-law) forbade me to move in a clumsy way. She told me to be careful about movement and timing of movement. **M: “Please explain to me.”** R: There are also fears of evil spirits. You cannot go out at noon, evening, dawn, and night. Evil spirits will catch you. (R25)

… They (aunts) talked to me. They told me how to move and what was good for me. They forbade me to go anywhere. They told me to be careful in my movement. They forbade me to go anywhere at noon. They told me to stay always in my room. (R06)

My mother told me to be careful. She forbade me to do heavy work. She forbade me to go outside at evening. I took fire (match box) with me wherever I went at noon or at night, at the time of Khon. (R23)

#### Emotional support and assurance

Emotional support and assurance refer to the provision of re-assurance and sympathetic listening that results in feelings of comfort and security. Women described emotional support as chatting about their comforts, discomforts, and the progression of their pregnancies. They referred to the people who consoled them and whom they confided in about concerns about pregnancy and childbirth. Mothers (48%), sisters-in-law (48%), and husbands (44%) were the three most frequently-mentioned sources of emotional support:

… she *(jal)* told me, ‘Do not get afraid, nothing will happen.’ When I felt pain she told me, ‘Do not worry. Nothing will happen.’ (R10)

**M: “Who consoled you?”** R: “I used to chat with my husband.” (R08)

Neighbours also provided emotional support and assurance:

My neighbour Farida Apa used to tell me, ‘do not be scared.’ She encouraged me. She told me, ‘You will get what Allah offers you’. (R08)

Notably, only 4% of the respondents reported that their mothers-in-law provided them with emotional support during pregnancy. This low ranking is consistent with the traditional dominant role a mother-in-law has over her daughter-in-law in patrilocal kinship systems (28). The imbalance of authority and power contributes to an indifferent or even hostile relationship.

#### Resources and material goods

Resources and material goods refer to the sharing or provision of goods, money, or tools. Women frequently mentioned husband's participation in securing monetary resources for healthcare, foods with increased nutritional value, transportation, and other related expenses. Husbands were the primary source of resources and material goods mentioned by 45% of the respondents, followed by mother's resources mentioned by 10% and mother-in-law's resources mentioned by 8% of the respondents. There was less variability in the sources providing resources and material goods compared to the other most frequently-mentioned types of support. The provision of monetary support by husbands and mothers was captured in the following quotes:

My husband used to ask me how I was. He queried my condition. He told me to let him know what I needed. He gave me money. (R18)

I took money from my mother (natal home). I did not take it from my marital house. (R10)

## DISCUSSION

The study provided current examples of the type, content, and source of social support during pregnancy grounded in a specific context of rural Bangladesh. The data indicate that women in Matlab perceive the receipt of eight distinct types of support and, on average, have 10 supportive relations in their networks. Although the sources varied by type of support provided, the role of marital and natal kins was prominent in all the narratives. Certain relationships were of particular importance and include the most frequently (overall)-mentioned sources—mothers, mothers-in-law, sisters-in-law, and husbands. All of these relations are kin-based and, with the exception of women's mothers, result from marriage. The types of support were discussed in terms of the helping activities they comprised. Specific findings relevant to future research and social support interventions are discussed below.

Practical help with routine daily activities was the most acknowledged type of support among the participants and appears to be more forthcoming in a woman's natal home. Women reported receiving help with milking cows, bringing water from the tubewell, washing clothes in the pond, cleaning and straining paddy, and childcare. In developing-county settings where economic resources are scarce, non-monetary support of this kind may represent the most reliably-available type of support. Women likely value practical help with routine activities because it allows time for the extra rest recommended during pregnancy and may, in turn, decrease the stress associated with poor pregnancy outcomes. Injury (i.e. back-pain, falls) associated with heavy household work may also be prevented. Interventions that solicit practical support from the family during pregnancy and promote a women's ability to spend time with her natal kins may foster maternal well-being. Further studies are needed to establish causal links between providing practical help in the context of a women's daily life with improvements in pregnancy outcomes.

The transcripts revealed the persistence of traditional advice regarding constraints on movement during pregnancy. The advice is reflective of the belief that pregnancy is a period of increased vulnerability to supernatural spirits. Decreased movement is a precautionary intervention aimed at preventing spirit attraction, particularly in the later stages of pregnancy. Previous research in Bangladesh has described restrictions of activity linked to beliefs in supernatural sprits (29, 30). With respect to health, researchers have noted that restrictions of mobility can reduce actual and potential access to needed medical services (31, 32). Interventions that seek to increase healthcare access should consider the receipt of messages that act as deterrents to women's mobility.

The documentation of the sources of emotional support and assurance revealed the unexpected role husbands play in supporting their wives. Typically, women are thought to provide substantially more emotional support than men due to their increased empathy (19, 33). Therefore, it is not surprising that mothers and sisters-in-law are the top sources mentioned by the women. In contrast, men are thought to lack emotional expressiveness and their contribution to emotional support often downplayed. However, our findings suggest that women perceive that their husbands do provide some degree of emotional support and assurance during pregnancy. A recent study by Singh and Ram in rural Ahmadnagar, India, also showed that a substantial proportion of men provided a notable level of support to their wives during pregnancy (34). Further investigation of the benefits associated with galvanizing spousal support during pregnancy is recommended.

Specific to the Matlab setting, CHRWs are instrumental in providing information and advice, particularly about where to deliver. Yet, although all the participants reported being visited by a CHRW during their pregnancies, only 60% mentioned them as sources of support. Aside from these specially-trained laywomen, the respondents did not list health professionals in their networks, suggesting that pregnant women do not consider professionals as sources of support. This finding corroborates other research that shows that non-professionals are the preferred and prominent social support providers (6). As social support appears to be a lay resource, interventions that encourage women to use and enhance personal networks are warranted.

A behaviour-change communication and social mobilization programme in Bangladesh provides some evidence that improvements in lay social support during pregnancy can be achieved. One of the programme goals was to generate or increase the support provided by husbands and mothers-in-law during pregnancy. The provision of positive support was highlighted through community mobilization efforts and a mass-media campaign. Family members were encouraged to make available regular and nutritious foods, allow women to take rest, and avoid heavy work. Social support was presented as a social custom to be followed during pregnancy. Post-evaluation data suggest that positive changes occurred in terms of the behaviour and attitudes of mothers-in-law towards their daughters-in-law and husbands towards their wives. Health workers were encouraged to continue community mobilization through group discussions about the need for the supportive role of husbands, mothers-in-law, and other members of the family during pregnancy (35). Replication of this approach deserves further consideration.

### Limitations

The findings must be considered within the scope of limitations of the study. As with all research involving self-reported interview responses, there is the potential for recall bias. Further, the study design did not allow for analysis of the need for support, the timing of support in relation to the stages of pregnancy, and satisfaction with the support received. The study also measured perceived versus received support. Perceived support, rather than received support, is most closely linked to health outcomes (36). Despite the fact that the study was carried out in Matlab, an area with sustained maternal and child-health programmatic inputs (e.g. CHRW's home visitation), the findings are likely reflective of women's support functions during pregnancy in other areas of Bangladesh—not in the sense of statistical generalization but in the sense of transferability of knowledge in ethnographic work.

### Conclusions

Specific and contextualized data from this study provide possible insights to researchers studying social support and programme staff developing interventions in this population. The potential to influence health outcomes positively depends on predicting which supportive functions will be most effective to a particular kind of stressor (37). It is likely that, in pregnancy, only certain types or sources of support may be helpful, such as practical help by members of a women's naturally-occurring social network. Formative qualitative research that details the nature of such support networks in distinct cultural settings is an important first step in establishing effective social support interventions for pregnant women.

## ACKNOWLEDGEMENTS

The study is a collaboration between the Center for Research on Maternal and Newborn Survival, Emory University, USA and the International Centre for Diarrhoeal Disease Research, Bangladesh, funded through the Woodruff Health Sciences Center Foundation grant to the Center for Research on Maternal and Newborn Survival, Nell Hodgson Woodruff School of Nursing, Emory University. In addition, a grant (No. F31NR010650) from the National Institute of Nursing Research further supported the first author. The content is solely the responsibility of the authors and does not necessarily represent the official views of the National Institute of Nursing Research, the National Institutes of Health, or the Woodruff Health Sciences Center Foundation.

The authors thank Allison Moran who facilitated local approvals to conduct the study.
